# Revolutionizing oncology care: pioneering AI models to foresee pneumonia-related mortality

**DOI:** 10.3389/fonc.2025.1520512

**Published:** 2025-03-19

**Authors:** Qunzhe Ding, Yi Zhang, Zihao Zhang, Peijie Huang, Rui Tian, Zhigang Zhou, Ruilan Wang, Yun Xie

**Affiliations:** ^1^ School of Information Management, Wuhan University, Wuhan, Hubei, China; ^2^ Department of Rheumatology and Immunology, Changzheng Hospital, Naval Military Medical University, Shanghai, China; ^3^ Georgetown University Medical Center Department of Oncology, Washington D.C., CO, United States; ^4^ Department of Critical Care Medicine, Shanghai General Hospital, Shanghai Jiao Tong University School of Medicine, Songjiang, Shanghai, China

**Keywords:** SEER, pneumonia, cancer, mortality, AI models

## Abstract

**Background:**

Pneumonia is a leading cause of morbidity and mortality among patients with cancer, and survival time is a primary concern. Despite their importance, there is a dearth of accurate predictive models in clinical settings. This study aimed to determine the incidence of pneumonia as a cause of death in patients with cancer, analyze trends and risk factors associated with mortality, and develop corresponding predictive models.

**Methods:**

We included 26,938 cancer patients in the United States who died from pneumonia between 1973 and 2020, as identified through the Surveillance, Epidemiology, and End Results (SEER) program. Cox regression analysis was used to ascertain the prognostic factors for patients with cancer. The CatBoost model was constructed to predict survival rates via a cross-validation method. Additionally, our model was validated using a cohort of cancer patients from our institution and deployed via a free-access software interface.

**Results:**

The most common cancers resulting in pneumonia-related deaths were prostate (n=7300) and breast (n=5107) cancers, followed by lung and bronchus (n=2839) cancers. The top four cancer systems were digestive (n=5882), endocrine (n=5242), urologic (n=5198), and hematologic (n=3104) systems. The majority of patients were over 70 years old (57.7%), and 54.4% were male. Our CatBoost model demonstrated high precision and accuracy, outperforming other models in predicting the survival of cancer patients with pneumonia (6-month AUC=0.8384,1-year AUC=0.8255,2-year AUC=0.8039, and 3-year AUC=0.7939). The models also revealed robust performance in an external independent dataset (6-month AUC=0.689; 1-year AUC=0.838; 2-year AUC=0.834; and 3-year AUC=0.828). According to the SHAP explanation analysis, the top five factors affecting prognosis were surgery, stage, age, site, and sex; surgery was the most significant factor in both the short-term (6 months and 1 year) and long-term (2 years and 3 years) prognostic models; surgery improved patient prognosis for digestive and endocrine tumor sites with respect to both short- and long-term outcomes but decreased the prognosis of urological and hematologic tumors.

**Conclusion:**

Pneumonia remains a major cause of illness and death in patients with cancer, particularly those with digestive system cancers. The early identification of risk factors and timely intervention may help mitigate the negative impact on patients’ quality of life and prognosis, improve outcomes, and prevent early deaths caused by infections, which are often preventable.

## Introduction

Pneumonia is a principal cause of infectious mortality worldwide, accounting for 2.5 million deaths in 2019 (1). While the burden of this disease predominantly affects elderly individuals and children under five years of age in developing countries, the recurrent emergence of new viruses over the past decades has reemphasized the importance of pneumonia as a public health risk (2). These challenges are even more pronounced in high-risk groups, particularly those who are immunosuppressed, such as patients undergoing active cancer treatment (3). There is growing interest in understanding the risk of viral pneumonia among cancer survivors (4–6). Patients with cancer are more susceptible to infections than the general population due to the immunosuppressive effects of various cancer therapies (7, 8). Previous studies have reported a greater risk of hospitalization and death due to pneumonia in patients with hematologic malignancies (9, 10). Patients receiving treatment for hematologic cancers are more vulnerable to infections due to severe deficiencies in both the innate and adaptive immune systems (11). Moreover, surgeries for cancers such as lung, esophageal, and head and neck cancers are highly invasive and can lead to serious postoperative complications, including pneumonia (12). Additionally, cancer patients often suffer from comorbidities induced by antitumor treatments, such as diabetes, dyslipidemia, hypertension, and obesity, which can contribute to pneumonia severity (9, 11, 13). The risk factors for various cancers are strikingly similar to those for the prognosis of pneumonia and other chronic diseases, including advanced age, smoking, poor diet, obesity, and alcohol consumption (14). A cancer diagnosis and antineoplastic treatments may be potential risk factors for severe pneumonia (15–19), although evidence for this presumed association is sparse.

Given this background, the risk of cancer survivors dying from pneumonia may be high, especially for those with lung, esophageal, head and neck cancers, and hematologic malignancies. Quantifying the broad impact of these infections on the prognosis of patients with cancer is crucial for raising awareness and allocating appropriate resources for prevention and treatment (20). Furthermore, from a public health and health policy perspective, identifying cancer patients at greater risk of dying from these infections is vital (21).

The Surveillance, Epidemiology, and End Results (SEER) database is among the largest and most extensively studied population-based cancer registry databases in the world. Owing to the information provided in the SEER database regarding the primary cancer site, cause, and time of death, which is linked with national mortality statistics in the United States, an assessment of deaths caused by pneumonia in cancer patients in the U.S. can be made. In this large-scale, population-based longitudinal study, we investigated the association between cancer incidence and the risk of death from pneumonia. Additionally, by leveraging deep learning, we established predictive models for pneumonia mortality in patients with cancer.

This study aimed to fill the existing gap in accurate predictive models for pneumonia-related mortality in patients with cancer. By employing advanced machine learning techniques, we seek to provide a more nuanced understanding of the risks and factors influencing pneumonia outcomes in cancer patients, ultimately contributing to improved clinical interventions and policymaking.

## Methods

### Database and data collection

The data for this study were extracted from the Surveillance, Epidemiology, and End Results (SEER) program of the National Cancer Institute, which covers approximately 28% of the U.S. population (2). The patient and disease characteristics recorded in the SEER database are generally considered representative of the entire U.S. population (3). Deaths due to pneumonia were defined via the SEER variable “Cause of Death (COD). Patient and disease characteristics, including age at diagnosis, race, income, education, urban versus rural residence, marital status, year of diagnosis, and treatment geographic region (SEER site), were collected from the SEER database. Survival time is measured in years from the time of cancer diagnosis to either death or the end of the follow-up period. For the purpose of the analysis, each factor was treated as a categorical variable.

The data used in the analysis were derived from the SEER 17 registries, encompassing tumor data from diagnoses made between 1973 and 2020. SEER*Stat software (version 8.3.5) was used to access the database. The case list eligibility criteria required that all cases had known ages and that all sites were recorded accordingly. All cases were defined via International Classification of Diseases for Oncology (ICD-O) histology codes. This study included all major tumor types, encompassing both benign and malignant neoplasms.

The inclusion criteria were as patients with the cause of the death of Pneumonia-Related Mortality. Exclusion criteria were as follows: (1) patients with unknown survival time; (2) patients with missing stage information; (3) patients with unknown surgery/radiation sequences; (4) patients with unknown primary cause of death; (5) patients missing grade records. The exclusion criteria, which removed patients with unknown information, were effective in ensuring data integrity and reducing bias in the survival analysis. This approach helped to create a more homogeneous cohort for accurate model training and validation.

Data regarding age, sex, grade, laterality, race, behavior (benign, borderline, *in situ*, or malignant), marital status, survival time, tumor site, and diagnosis date were obtained from the SEER database.

The SEER data are divided into test sets and internal validation sets at a ratio of 7:3. The external validation data were obtained from Shanghai General Hospital.

### Ethical standards

The study adhered to medical ethical standards and was approved by the Medical Ethics Committee of Shanghai General Hospital [Approval No. [2021]KY041)]. Patient confidentiality was maintained by anonymizing the data, and only hospitalization numbers were used for data validation.

### Incidence-based mortality rate calculation

The incidence-based mortality rate attributable to pneumonia (IBMR) for the different cancers was calculated. Joinpoint regression was used to assess the temporal trends in IBMR due to pneumonia, which involved fitting a series of joined straight lines on a log scale to the annual age-adjusted rates and quantifying them via the annual percentage change (APC).

### Cox proportional hazards model

To evaluate the independent impact of patient and disease characteristics on pneumonia-specific death (SSD), a Cox proportional hazards model was constructed using the following covariates in test datasets: age, race, age, marital status, surgery performed, chemotherapy, radiation, grade, stage, PRCDA, surgery and radiation sequence, tumor site record, number of primary tumors, type of reporting source, and first malignant primary tumor indicator. The first malignant primary tumor indicator was removed because it was not a significant predictive factor, as indicated by its nonsignificant p value. The model also included an *a priori* assessment of the first-order interactions between surgical techniques and all other independent variables included in the model.

### Statistical analysis

All the statistical tests were two-sided, and the significance level was set at p < 0.05. The analyses were performed via SEER*Stat 8.1.5 (http://seer.cancer.gov/seerstat/), Joinpoint 4.1.1.1 (http://surveillance.cancer.gov/joinpoint/), and SAS 9.3 (Cary, North Carolina).

This comprehensive approach to data collection and analysis ensures a robust examination of the relationship between cancer and pneumonia-related mortality, providing a foundation for the development of predictive models that can inform clinical decision-making and patient care.

The experimental analyses were conducted via Python version 3.10.9, leveraging key libraries, including pandas for data manipulation, NumPy for numerical operations, and Scikit-Learn for model implementation. Patients were randomly allocated into the training and testing cohorts at a 7:3 ratio, with approximately 70% of the dataset dedicated to training and the remaining 30% dedicated to validation. The optimal hyperparameters were determined through ten-fold cross-validation during the training phase. The predictive performance of the CatBoost algorithm was rigorously compared with that of established machine learning models such as logistic regression (LR), support vector machine (SVM), random forest (RF), XGBoost, gradient boosting machine (GBM), and LightGBM.

Unlike traditional statistical methods such as Cox regression, which assume a log-linear relationship between covariates and the hazard function, novel machine learning models such as CatBoost are non-parametric and capable of capturing complex, non-linear relationships and higher-order interactions among features. This flexibility enables CatBoost to model survival data without relying on pre-specified assumptions about the effects of covariates.

Model efficacy was evaluated via receiver operating characteristic (ROC) curve analysis, with a focus on the area under the ROC curve (AUC) and confusion matrices as principal evaluative metrics. In addition, we employed the SHAP method to enhance our understanding of the model’s decision-making process, providing insights into how features impact the model’s predictions. This analysis aids in interpreting complex model behaviors and ensuring the transparency and reliability of our findings.

### CATBOOST model

Introduced by Yandex in 2017 (27), the CatBoost algorithm was designed to efficiently handle categorical data while improving the robustness and accuracy of gradient boosting methods. It employs ordered boosting, a unique strategy that mitigates overfitting by leveraging randomized permutations of the dataset during tree construction. Unlike conventional gradient boosting methods, which may introduce target leakage when encoding categorical variables, CatBoost processes categorical features natively, reducing the need for extensive preprocessing. CatBoost utilizes oblivious decision trees, where each level of the tree applies the same splitting criterion across all nodes. This structural constraint enhances computational efficiency and reduces overfitting, making the model particularly well-suited for datasets with complex categorical structures. The CatBoost model is trained as an ensemble of decision trees using the following formulation:


$$\text{Z }=\;\sum_{\text{n}=1}^{N}\alpha_{n}\;H_{n}\;\left(x_{i}\right)$$

In this formulation, Z represents the predicted risk score for a given patient i , indicating the likelihood of an event occurring over time in survival analysis. N is the total number of decision trees in the ensemble. $H_{n}\left(x_{i}\right)$ is the prediction output of the n -th oblivious decision tree, which is a decision function mapping input feature $x_{i}$ to an estimated probability. $\alpha_{n}$ is the weight assigned to the n -th tree, determining its contribution to the final prediction. Each decision tree $H_{n}\left(x_{i}\right)$ in CatBoost is constructed using ordered boosting, a unique technique that reduces target leakage and improves generalization. Unlike conventional gradient boosting, which may introduce bias by using the same dataset to train and construct trees, ordered boosting ensures that each tree learns from properly permuted past observations, maintaining independence in predictions. This strategy enhances model robustness and reduces overfitting.

CatBoost further distinguishes itself by employing oblivious decision trees, where each level of the tree applies the same splitting rule across all nodes. This structural constraint simplifies decision pathways and improves computational efficiency, particularly for datasets rich in categorical features. By iteratively refining tree structures and weight coefficients, the model achieves an optimal balance between accuracy and efficiency.

## Results

### Clinical characteristics of patients

**Figure 1:**
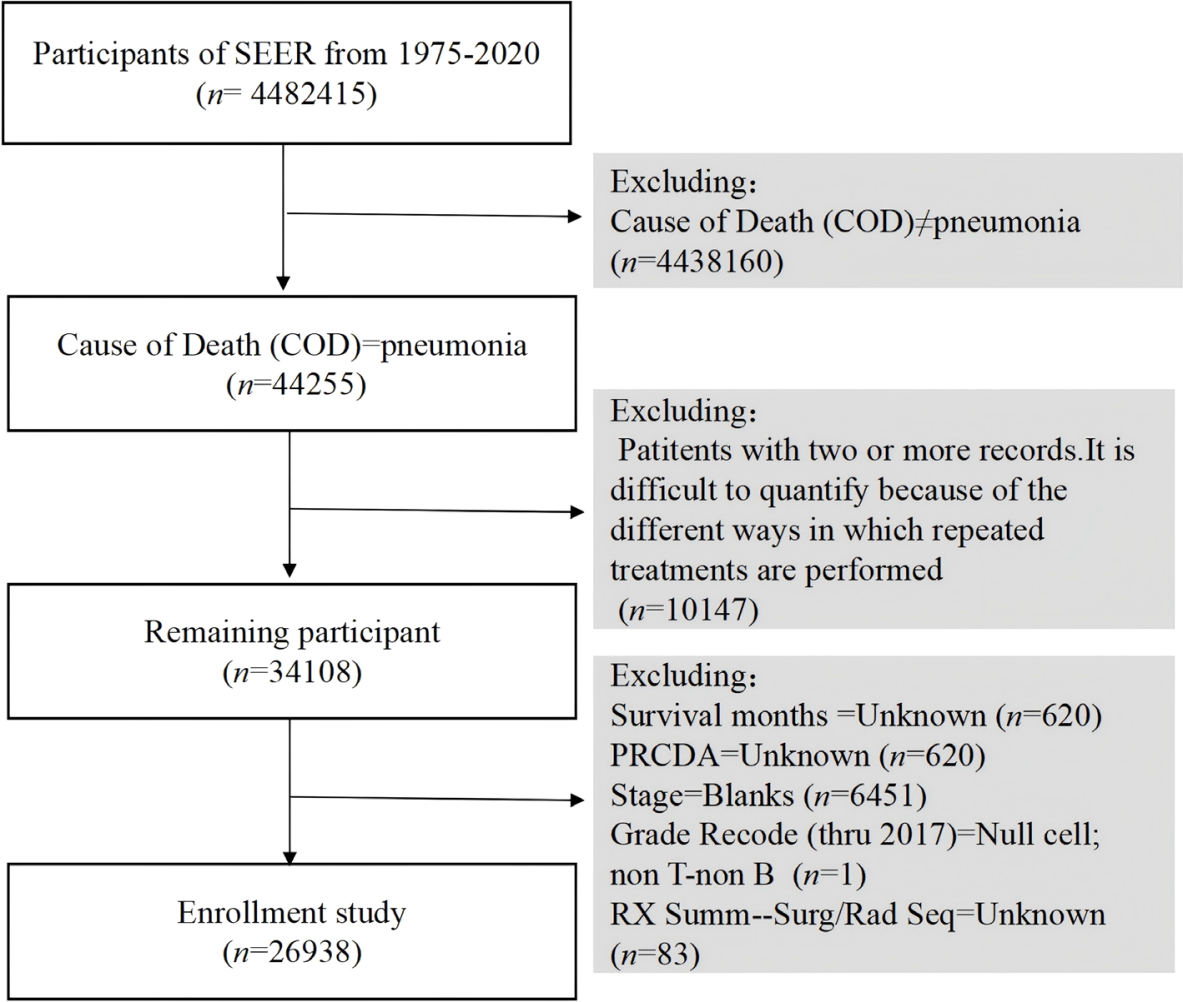
Flowchart of the research.

Understanding the demographic and clinical characteristics of cancer patients who develop pneumonia is crucial for identifying high-risk groups and tailoring interventions. In our analysis of data from the SEER database spanning 1975 to 2020, we identified 4,482,415 cancer diagnosis cases, among which 44,255 patients died from pneumonia during the study period (**Figure 1**).

After applying the exclusion criteria, the final study cohort comprised 26,938 patients. The demographic distribution revealed 47.5% males, with 86.4% of the patients being Caucasian. The 80 years and above age group was the largest group, accounting for 33.7%, followed by the 70–79 years age group, accounting for 33.5%. Regarding marital status, 48.9% of the patients were married, whereas 40.8% were categorized as widowed/divorced/other. In terms of treatment, 13.6% of patients received chemotherapy, 66.6% underwent surgery, and 79.2% received radiation therapy. The majority of patients were diagnosed with Grade II tumors (28.7%), with Grades I, III, and IV representing 11.1%, 15.6%, and 2.8%, respectively. B-cell and T-cell neoplasms accounted for 2.9% and 0.3% of the cases, respectively.

Among all patients who died after a cancer diagnosis, pneumonia-related deaths constituted 0.6% of all mortalities (26,938 of 4,482,415). Notably, 21.8% of these deaths were attributed to digestive system tumors, followed by endocrine tumors (19.4%). **Table 1** details the characteristics of the patients who died of pneumonia.

**Table 1:** Baseline characteristics of patients included from SEER data cohort.

Characteristic		Case(*n*=26938)	%
Sex	Male	12801	0.475202316
	Female	14137	0.524797684
Race	White	23268	0.863761229
	Black	2412	0.089538941
	Other	1258	0.046699829
Age	<60	3423	0.127069567
	60-69	5418	0.201128517
	70-79	9024	0.334991462
	80+	9073	0.336810454
Marital Status	Married	13169	0.488863316
	Single	2763	0.102568862
	Widow/divorced/other	11006	0.408567822
Surgery performed	Not performed	9005	0.334286139
	Performed	17933	0.665713861
Chemotherapy	No	23275	0.864021085
	Yes	3663	0.135978915
Radiation	No	5597	0.207773406
	Yes	21341	0.792226594
Grade	Grade I	2990	0.11099562
	Grade II	7723	0.286695375
	Grade III	4204	0.156062068
	Grade IV	747	0.027730344
	B-cell	793	0.029437969
	T-cell	79	0.00293266
	Unknow	10402	0.386145965
Stage	Unstaged	4240	0.157398471
	Localized	11781	0.43733759
	Regional	5450	0.20231643
	Distant	3189	0.118382953
	Localized/regional (Prostate cases)	2278	0.084564556
PRCDA	No	15090	0.560175217
	Yes	11848	0.439824783
Time_from_diagnosis_to_treatment	0 days	9918	0.368178781
	1-30 days	1532	0.056871334
	31-60 days	777	0.028844012
	61-120 days	313	0.011619274
	Unknown	14398	0.534486599
RX_Summ:Surg_or_Rad_Seq	No radiation and/or no surgery; unknown if surgery and/or radiation given	23835	0.884809563
	Radiation after surgery	2688	0.099784691
	Radiation prior to surgery	415	0.015405747
Site recode ICD O3	Urological	5198	0.192961616
	Digestive	5882	0.218353256
	Endocrine	5242	0.194594996
	Hematologic	3104	0.11522756
	Respiratory	2072	0.076917366
	Reproductive	1904	0.070680823
	Other	3536	0.131264385
Sequence_number	One primary only	25109	0.932103348
	2nd of 2 primaries	1239	0.045994506
	3rd of 3 or more primaries or more	590	0.021902146
Type of Reporting Source	Hospital inpatient/outpatient or clinic	25799	0.957717722
	Laboratory only	584	0.021679412
	Physicians office	378	0.014032222
	Other	177	0.006570644
First malignant primary indicator	No	1095	0.040648897
	Yes	25843	0.959351103

A total of 9,918 patients (36.8%) received immediate medical intervention, whereas 777 patients (2.9%) received treatment for more than one month after tumor diagnosis.

### Univariate and multivariate cox regression analyses

Identifying significant predictors of survival in cancer patients with pneumonia is essential for developing accurate predictive models. Univariate Cox regression analysis was conducted to identify variables that significantly affected overall survival (OS) and cancer-specific survival (CSS) in cancer patients with pneumonia in the test datasets. The variables included age at diagnosis, race, marital status, histological type, number of months from diagnosis to treatment, grading, and treatment information (**Table 2**).

**Table 2:** Multivariate analysis of the hazard ratio for death from pneumonia in patients diagnosed with cancer (1979–2020).

Multivairate COX BASS		HR	95% CI	P-value
Sex	Male			
	Female	0.739757441	(0.7168, 0.7635)	0.00000
Race	White			
	Black	1.329761758	(1.2551, 1.4089)	0.00000
	Other	0.959110921	(0.9182, 1.0019)	0.06055
Age	<60			
	60-69	1.562379036	(1.4933, 1.6347)	0.00000
	70-79	2.463702039	(2.3559, 2.5764)	0.00000
	80+	4.493775089	(4.2843, 4.7135)	0.00000
Marital Status	Married			
	Single	1.309297647	(1.2558, 1.3650)	0.00000
	Widow/divorced/other	1.237970213	(1.2036, 1.2733)	0.00000
Surgery performed	Not performed			
	Performed	0.563993836	(0.5400, 0.5890)	0.00000
Chemotherapy	No			
	Yes	1.096231182	(1.0549, 1.1391)	0.00000
Radiation	No			
	Yes	0.794788193	(0.7577, 0.8336)	0.00000
Grade	Grade I			
	Grade II	1.065490836	(1.0203, 1.1126)	0.00409
	Grade III	1.221102589	(1.1633, 1.2817)	0.00000
	Grade IV	1.271817497	(1.1722, 1.3799)	0.00000
	B-cell	1.093462145	(0.9981, 1.1980)	0.05505
	T-cell	1.598005235	(1.2715, 2.0083)	0.00006
	Unknow	1.017477244	(0.9740, 1.0629)	0.43685
Stage	Unstaged			
	Localized	0.645162327	(0.6156, 0.6761)	0.00000
	Regional	0.776043958	(0.7378, 0.8163)	0.00000
	Distant	1.489477403	(1.4189, 1.5636)	0.00000
	Localized/regional (Prostate cases)	0.473976117	(0.4429, 0.5073)	0.00000
PRCDA	No			
	Yes	1.043223697	(1.0176, 1.0695)	0.00086
RX_Summ:Surg_or_Rad_Seq	No radiation and/or no surgery; unknown if surgery and/or radiation given			
	Radiation after surgery	1.327904914	(1.2482, 1.4128)	0.00000
	Radiation prior to surgery	1.136934325	(1.0227, 1.2639)	0.01751
Site recode ICD O3	Urological			
	Digestive	1.072260143	(1.0236, 1.1233)	0.00325
	Endocrine	0.989309978	(0.9384, 1.0429)	0.68991
	Hematologic	0.678821185	(0.6353, 0.7253)	0.00000
	Respiratory	1.78191288	(1.6780, 1.8922)	0.00000
	Reproductive	0.84339386	(0.7913, 0.8989)	0.00000
	Other	1.168454044	(1.1088, 1.2313)	0.00000
Sequence_number	One primary only			
	2nd of 2 primaries	0.969667296	(0.8558, 1.0986)	0.62879
	3rd of 3 or more primaries or more	0.711955951	(0.6553, 0.7735)	0.00000
Type of Reporting Source	Hospital inpatient/outpatient or clinic			
	Laboratory only	0.755579252	(0.6939, 0.8227)	0.00000
	Physicians office	0.930715171	(0.8390, 1.0325)	0.17508
	Other	1.296874363	(1.1174, 1.5052)	0.00062
First malignant primary indicator	No			
	Yes	0.929671522	(0.8142, 1.0615)	0.28099

Multivariate Cox regression analysis was subsequently performed to control for confounding factors and reveal independent predictors of OS and CSS. The results indicated that female sex, black race, age over 60 years, Grade III or IV tumors, T-cell type, and three or more primaries were significantly associated with poorer OS and CSS. In terms of treatment, multivariate Cox regression analysis revealed that surgery, chemotherapy, and radiation therapy could prolong OS and CSS. Prognosis was also influenced by societal factors, including marital status, with marriage being significantly correlated with higher survival rates.

### Establishment and evaluation of predictive models

Developing robust predictive models for pneumonia-related mortality in cancer patients can significantly enhance clinical decision-making and patient care. On the basis of the results obtained, we developed a CatBoost predictive model to predict the survival of cancer patients with pneumonia at six months, one year, two years, and three years. The patients were divided into a training dataset and a test dataset at a 7:3 ratio. To ensure model stability, tenfold cross-validation was employed in the training set for iterative testing and tuning, which allowed us to determine key hyperparameters and generate the optimal model (**Table 3**). The final model was then evaluated on the test set, where we calculated the corresponding AUC values for each model in different survival period (**Table 4**).

**Table 3:** The optimal parameters of the Catboost model.

Parameter	Value
Learning rate	0.01
Iteration	1000
Depth	6
L2 leaf reg	5
Border count	128
Score_function	L2

**Table 4:** Prognostic Model of death from pneumonia in patients diagnosed with cancer (1979–2020).

	6-month survival	1-year survival	2-year survival	3-year survival
LR	0.7626	0.7428	0.7289	0.7211
RF	0.7972	0.7848	0.7603	0.7458
SVM	0.8215	0.8120	0.7864	0.7751
Xgboost	0.8372	0.8250	0.8023	0.7931
GBM	0.8381	0.8252	0.8025	0.7923
LightGBM	0.8369	0.8216	0.8037	0.7926
Catboost	0.8384	0.8255	0.8039	0.7939

**Figure 2:**
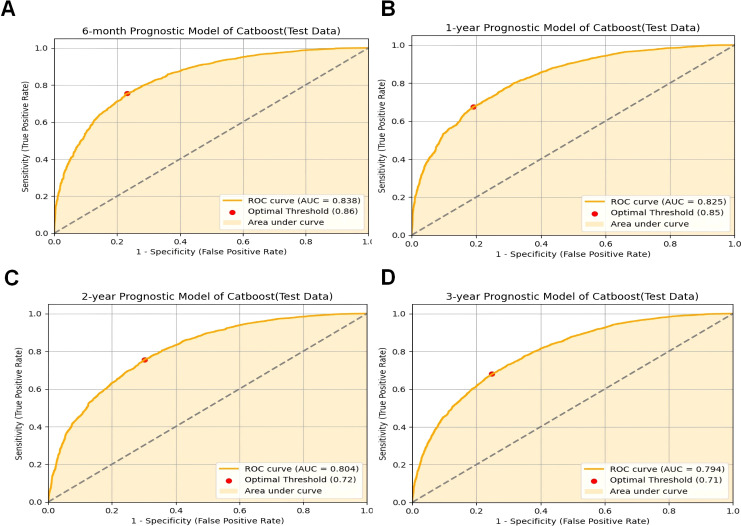
CatBoost model evaluation. **(A)** ROC curve for the 6-month prognostic model (test data); **(B)** ROC curve for the 1-year prognostic model (test data); **(C)** ROC curve for the 2-year prognostic model (test data); **(D)** ROC curve for the 3-year prognostic model (test data);ROC receiver operating characteristic curve; AUC area under the curve; CatBoost categorical boosting.

The CatBoost model demonstrated excellent performance in predicting the survival of cancer patients with pneumonia at six months (AUC = 0.8384 in the test set), one year (AUC = 0.8255), two years (AUC = 0.8039), and three years (AUC = 0.7939) (**Figure 2**). Compared with traditional machine learning algorithms, the CatBoost model exhibited superior or comparable performance across all timeframes (**Table 4**). For example, at six months, CatBoost achieved an AUC of 0.8384, slightly outperforming XGBoost (AUC = 0.8372), GBM (AUC = 0.8381), and LightGBM (AUC = 0.8369). Similarly, at one year, CatBoost reached an AUC of 0.8255, higher than LightGBM (AUC = 0.8216) and on par with GBM (AUC = 0.8252). Over the two- and three-year timeframes, CatBoost continued to slightly outperform its counterparts, including XGBoost, GBM, and LightGBM. Traditional models such as logistic regression (LR), random forest (RF), and support vector machines (SVM) generally exhibited lower AUC values, ranging from 0.7626 to 0.8215 at six months and decreasing over longer timeframes. Notably, all models demonstrated a decreasing trend in predictive performance over longer timeframes, with AUC values gradually declining from six months to three years. This trend suggests that as the follow-up period extends, the prediction task becomes more challenging, likely due to increased variability in clinical factors, treatment responses, and disease progression. While CatBoost consistently outperformed or matched other models, its predictive ability also declined over time, highlighting the inherent difficulty in long-term survival prediction for cancer patients with pneumonia.

### External validation of the model

**Figure 3:**
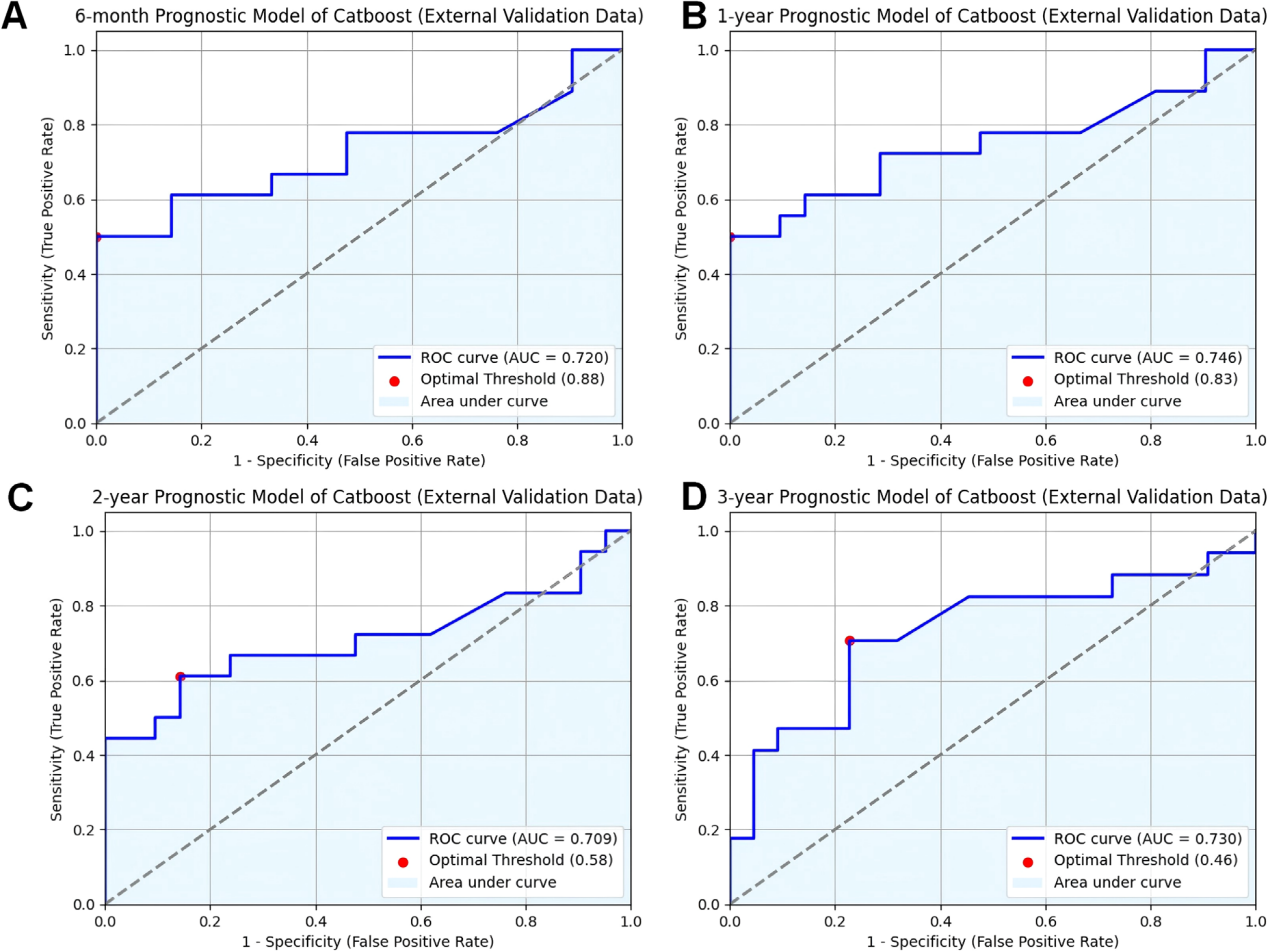
Validation of CatBoost models from external database. **(A)** ROC curve for the 6-month prognostic model (external validation data); **(B)** ROC curve for the 1-year prognostic model (external validation data); **(C)** ROC curve for the 2-year prognostic model (external validation data); **(D)** ROC curve for the 3-year prognostic model (external validation data); ROC receiver operating characteristic curve; AUC area under the curve; CatBoost categorical boosting.

Validating predictive models in external datasets is essential to assess their generalizability and reliability in real-world clinical settings. To assess the reliability and generalizability of the model, we conducted external validation using clinical and prognostic data from 38 cancer patients at our institution. The CatBoost model demonstrated strong predictive performance in this independent dataset, achieving AUC values of 0.689 at six months (**Figure 3A**), 0.838 at one year (**Figure 3B**), 0.834 at two years (**Figure 3C**), and 0.828 at three years (**Figure 3D**). These results indicate that the model maintains consistent performance across different time intervals, supporting its applicability in real-world clinical settings.

**Figure 4:**
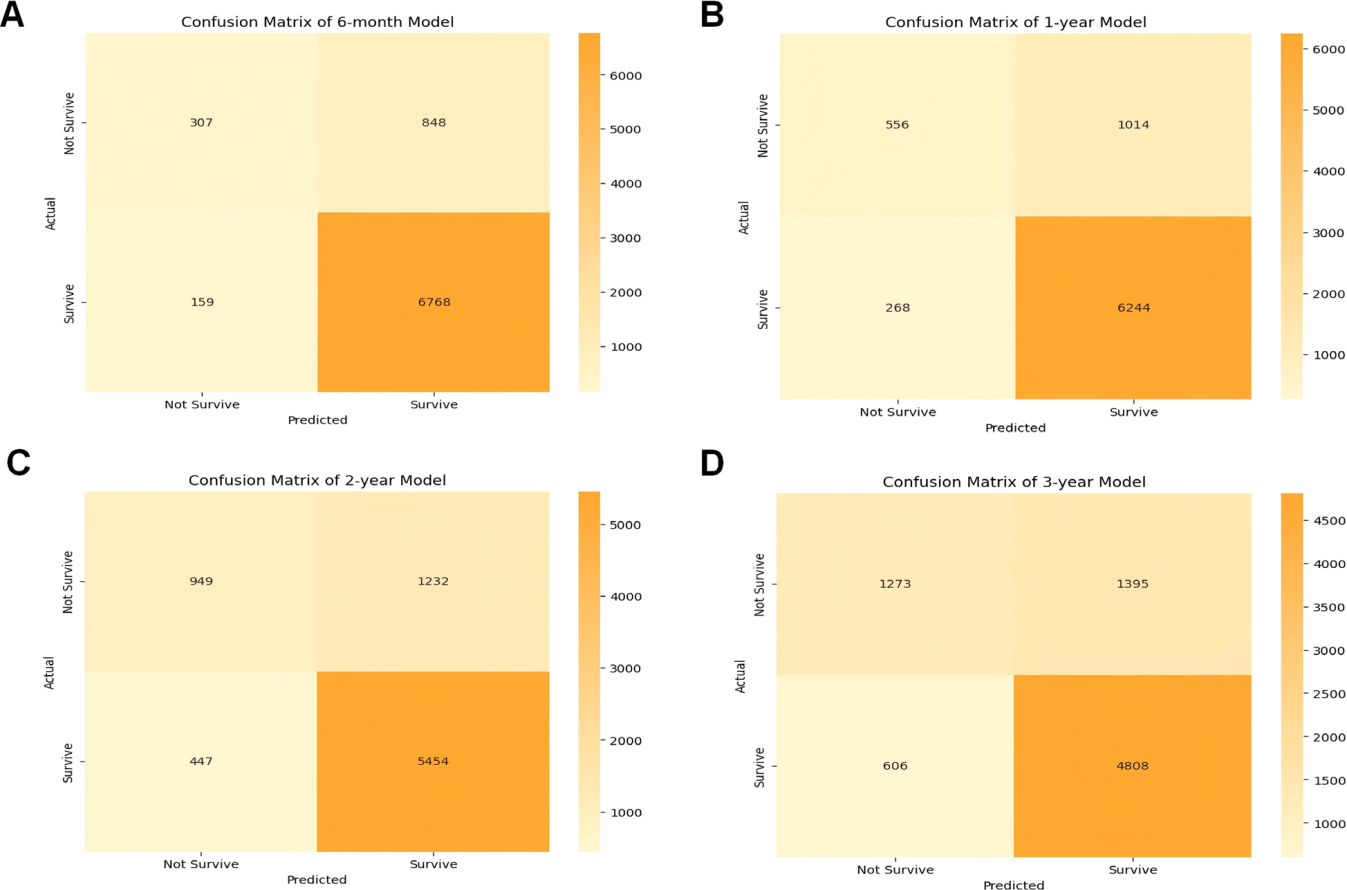
Confusion matrix of the CatBoost model’s predicted results in the test data. **(A)** Confusion matrix in the 6-month prognostic model; **(B)** confusion matrix in the 1-year prognostic model; **(C)** confusion matrix in the 2-year prognostic model; **(D)** confusion matrix in the 3-year prognostic model. TP true positive, TN true negative.

The effectiveness and accuracy of the CatBoost model were also evaluated via confusion matrix. The 6-month survival prediction model had an accuracy of 0.66 and a precision of 0.89 (**Figure 4A**); the 1-year survival model had an accuracy of 0.67 and a precision of 0.86 (**Figure 4B**); and the 2-year survival model had an accuracy of 0.68 and a precision of 0.82 (**Figure 4C**). The 3-year survival model had an accuracy of 0.68 and a precision of 0.77 (**Figure 4D**). Overall, our model was efficient and performed well. However, similar to the AUC trend, both precision and accuracy exhibited a slight decline over longer timeframes, which may be attributed to increased variability in patient outcomes and disease progression over time. Therefore, models predicting longer-term survival may be more limited in performance compared to short-term models. Despite this, the CatBoost model maintained relatively stable performance, demonstrating its robustness in survival prediction for cancer patients with pneumonia.

### SHAP analysis

Understanding the relative contributions of demographic and clinical factors in predicting pneumonia-specific mortality is crucial for developing personalized care plans. To evaluate the relative contributions of demographic and clinical factors in predicting pneumonia-specific mortality, we employed SHAP importance plots to analyze the best-performing CatBoost model. SHAP is a widely used novel explainability method for machine learning models that quantifies the impact of each feature on the model’s predictions. It assigns an importance score to each feature, representing the average magnitude of its contribution to the model’s output. This analysis not only identifies the most influential features but also provides insights into the decision-making process of the machine learning model.

**Figure 5:**
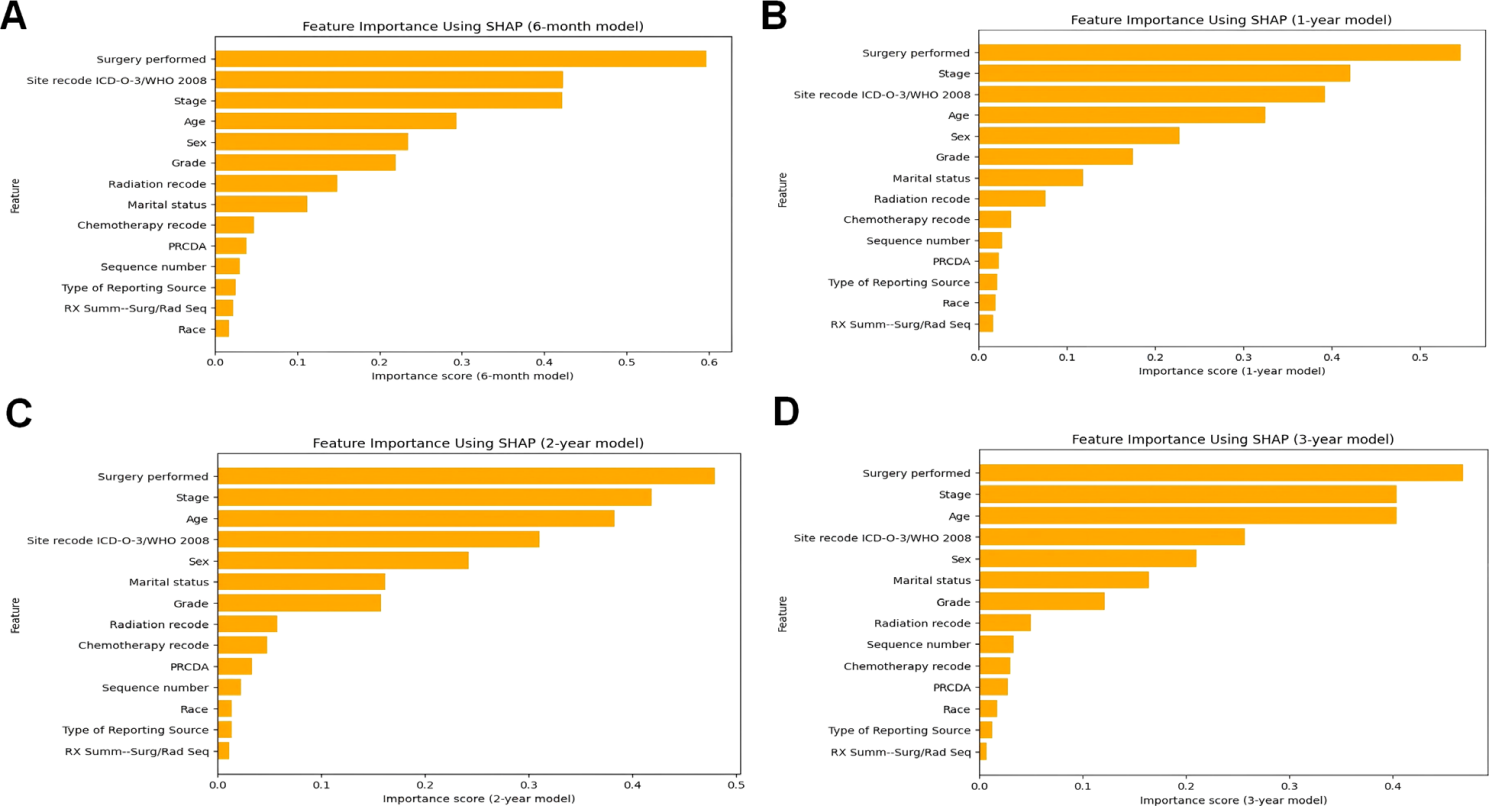
The ranking of clinical characteristics in terms of importance in the CatBoost prognostic model. **(A)** The ranking of clinical characteristics in terms of importance in the 6-month prognostic model; **(B)** The ranking of clinical characteristics in terms of importance in the 1-year prognostic. Model; **(C)** Ranking of clinical characteristics in terms of importance in the 2-year prognostic model; **(D)** Ranking of clinical characteristics in terms of importance in the 3-year prognostic model.

**Figures 5A–D** illustrates the SHAP importance plots for the 6-month, 1-year, 2-year, and 3-year predictive models, respectively. Across all timeframes, clinical factors consistently dominate the predictions, highlighting their critical role in assessing pneumonia-related mortality risk. Surgery performed, a clinical intervention, emerges as the most significant predictor across all models, underscoring its profound influence on patient outcomes. Tumor stage, another key clinical factor, consistently ranks as the second most important variable in the 1-year, 2-year, and 3-year predictive models, reflecting the direct association between cancer progression and pneumonia risk. Site recode ICD-O-3/WHO 2008, which represents cancer site, also ranks among the most influential features across all timeframes, further emphasizing the importance of clinical factors in determining patient vulnerability to pneumonia.

Demographic factors, while less influential in importance compared to clinical factors, still contribute meaningfully to the predictive models. Age, a critical demographic factor, ranks within the top four features across all models, affirming its significant impact on pneumonia mortality risk. Sex, though ranked lower than most clinical factors, exhibit consistent importance, suggesting that these demographic characteristics also influence patient outcomes.

Notably, the contribution of radiation and chemotherapy-related clinical factor, while evident, is less prominent than surgical intervention and tumor stage. This could be attributed to the variability in treatment regimens and patient response, which warrants further investigation in future studies.

In summary, the SHAP analysis shown in **Figure 5** reveals a clear pattern: clinical factors, particularly those related to surgical interventions, cancer progression, and cancer site, are the primary drivers of pneumonia-specific mortality predictions. Demographic factors, although less influential, still play a notable role, particularly age and marital status. These findings underscore the multifaceted nature of pneumonia mortality risk in cancer patients and highlight the importance of integrating both clinical and demographic factors into predictive models for personalized care.

### Further exploration of surgical impact using SHAP interaction plots

Understanding how specific clinical interventions, such as surgery, impact patient prognosis can inform targeted interventions and improve patient outcomes. In the previous section, using the SHAP importance plot, this study identified the key features that significantly influence patient prognosis. To further analyze how these features impact the model’s predictions, we employed the SHAP Summary Plot to explore the relationship between each specific feature and the predicted outcomes.

The SHAP summary plot visualizes the distribution of each feature’s impact on the model’s predictions. The color gradient provides insights into how variations in feature values influence the predicted outcome: red represents higher feature values, while blue corresponds to lower values. Points farther from the baseline SHAP value of zero indicate a stronger effect on the model’s output. This visualization offers a clearer understanding of the relationship between each feature and its SHAP value, providing valuable insights into how changes in feature values affect the predicted results.

**Figure 6:**
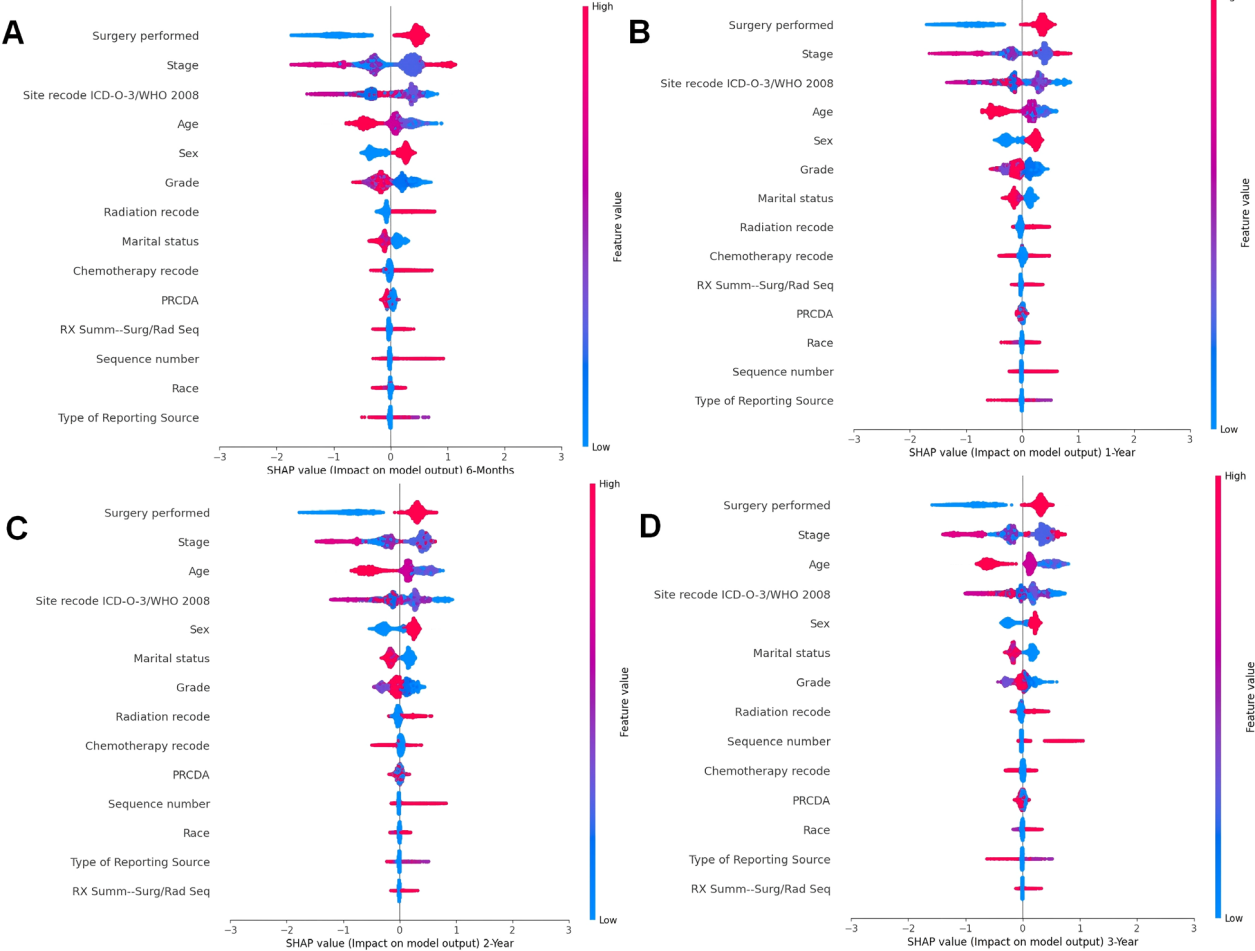
Summary beeswarm plot of features from SHAP importance analysis based on CatBoost model. **(A)**. The SHAP Importance Analysis in the 6-month prognostic model; **(B)** The SHAP Importance Analysis in the 1-year prognostic model; **(C)** The SHAP Importance Analysis in the 2-year prognostic model; **(D)** The SHAP Importance Analysis in the 3-year prognostic model.

Across all timeframes (**Figure 6**), Surgery performed emerges as the most influential feature in survival predictions. Positive SHAP values (red points) indicate that certain types of surgeries significantly improve survival probabilities, whereas negative SHAP values (blue points) suggest that specific surgeries may have a detrimental effect on survival. This consistent influence underscores the critical role of surgical intervention in determining survival outcomes for cancer patients with pneumonia.

Stage, another key clinical factor, is consistently ranked as one of the top predictors of survival. However, the SHAP summary plot does not reveal a clear trend in how this feature affects survival, reflecting the complex relationship between cancer staging and patient outcomes. Advanced cancer stages (typically associated with higher feature values) are often linked to poorer survival, but the impact can vary depending on other factors, such as treatment and patient characteristics.

The Site recode ICD-O-3/WHO 2008 variable shows that cancer location significantly influences survival predictions. The SHAP values suggest that cancers originating from lower-coded sites, such as Urological and Digestive systems, contribute more positively to survival outcomes. This is evidenced by higher SHAP values (red points) for these sites compared to others. Conversely, cancers from higher-coded sites demonstrate relatively lower SHAP values, indicating a lesser contribution to survival.

The Sex feature exhibits a consistent pattern across all time periods. Female patients (coded as 1, red points) are generally associated with positive SHAP values, indicating positively contribute survival probabilities. In contrast, male patients (coded as 0, blue points) tend to show lower SHAP values, suggesting a potential negative impact on survival outcomes. This observation aligns with known biological and behavioral differences, which may influence disease progression and response to treatment.

Age is another prominent demographic factor influencing survival predictions. Older patients (higher feature values, red points) are associated with lower survival probabilities (negative SHAP values), while younger patients (lower feature values, blue points) show positive contributions to survival. This pattern is consistent across all timeframes, highlighting the vulnerability of older patients to pneumonia-related complications.

In the previous section, we revealed the key features that significantly influence patient prognosis, among which surgery was the most important in all the models. Therefore, to further explore how surgery affects patient prognosis, we applied the SHAP interaction plot.

**Figure 7:**
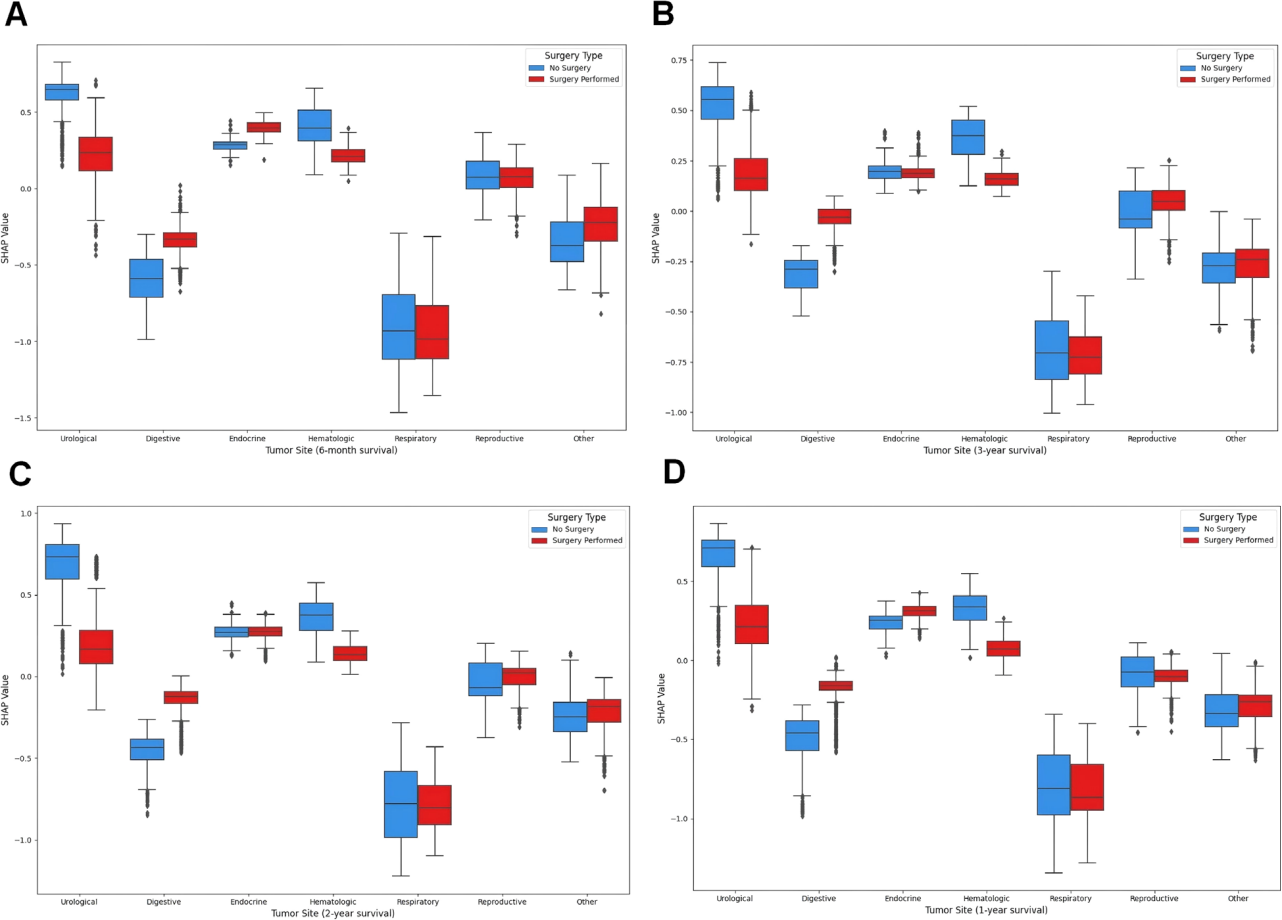
SHAP interaction plot. **(A)** The SHAP interaction plot of the 6-month prognostic model; **(B)** The SHAP interaction plot of the 1-year prognostic model; **(C)** The SHAP interaction plot of the 2-year prognostic model; **(D)** The SHAP interaction plot of the 3-year prognostic model.

By selecting combination of the features of disease site (Site recode ICD-O-3/WHO 2008) and Surgery performed, we attempted to determine whether surgeries at different sites have varying impacts on patient prognosis. In the interaction plot, the x-axis represents different disease sites, and the color scale indicates whether surgery was performed (red) or not (blue), with higher SHAP values indicating beneficial effects on outcomes, often leading to better prognosis. The shap interaction plot (**Figure 7**) revealed distinct patterns in the impact of surgery across different tumor sites. For urological, hematologic, and reproductive tumors, no-surgery cases showed higher SHAP values, indicating a greater contribution to survival. In contrast, digestive and other tumors had significantly higher SHAP values in surgery cases, suggesting improved prognosis with surgical intervention. Endocrine and respiratory tumors showed minimal differences between surgery and no-surgery groups, indicating limited impact of surgery on survival.

### Web-based model development

Developing accessible and user-friendly tools for clinicians and researchers can enhance the practical application of predictive models in clinical settings. We developed web-based applications to facilitate the utilization of our prognostic models by researchers and clinicians. (http://1.92.110.6:8091/, http://1.92.110.6:8092/, http://1.92.110.6:8093/, http://1.92.110.6:8094/). The Web-based applications enable the input of clinical characteristics for a new sample. Subsequently, the application processes this information to predict survival probabilities and to determine the survival status of the patient on the basis of the provided clinical data.

### Clinical implications of findings

Our study’s findings offer valuable insights for improving the management of cancer patients at risk of pneumonia-related mortality. The CatBoost model’s high accuracy in predicting survival probabilities across different time intervals enables early identification of high-risk patients and supports timely interventions. The SHAP analysis highlights the importance of surgical intervention, cancer stage, and tumor site in determining patient prognosis, emphasizing the need for personalized treatment plans. These insights can optimize healthcare resource allocation and improve patient outcomes. To facilitate practical application, we have developed web-based applications that allow clinicians to input patient-specific data and receive survival probability predictions. These tools provide a user-friendly interface for generating survival status predictions based on clinical data. Future research should focus on enhancing model accuracy and validating it across diverse populations to advance personalized cancer care.

## Discussion

The intersection of cancer and pneumonia presents a formidable challenge in patient care, with the mortality rate being a significant concern. The application of machine learning (ML) models to predict the prognosis of death due to pneumonia in cancer patients is a novel and promising development in this domain. Our study, which utilized data from the SEER database spanning nearly half a century, identified key trends and prognostic indicators that can inform the development of predictive models.

Kanayama et al. (2020) (22) and Abdel-Rahman (2020) (23) focused on the risk factors associated with pneumonia-related mortality in cancer patients. These findings emphasize the need for a deeper understanding of the mechanisms linking cancer, treatment modalities, and the propensity to develop severe pneumonia.

The demographics of our enrolled patients reflected the typical characteristics of the at-risk population, with a notable predominance of elderly and Caucasian individuals. These demographic data, coupled with the various treatment modalities received by patients, underscore the heterogeneity of the cancer patient population and the need for personalized predictive models. Our analysis revealed that clinical factors such as tumor grade, stage, and treatment modalities had a stronger influence on pneumonia-specific mortality compared to demographic factors like age and marital status. However, demographic factors still played a significant role, particularly in older patients and those with specific racial backgrounds.

A national analysis of complications associated with cancer treatment in the emergency room and inpatient settings revealed that advanced age, male sex, sepsis, pneumonia, and myocardial infarction were associated with hospitalization, whereas sepsis, myocardial infarction, and pneumonia were associated with inpatient mortality. The rate of emergency room visits for complications of systemic or radiation therapy has increased 5.5-fold in 10 years (24). Our findings indicate that pneumonia accounts for a small but significant proportion of all deaths among patients with cancer, emphasizing the need for targeted interventions. Cox regression analyses revealed several factors significantly associated with pneumonia-specific survival in patients with cancer, including age, race, marital status, histological type, time to treatment, grade, and treatment information. These factors, particularly the impact of surgical intervention, chemotherapy, and radiation therapy on survival outcomes, highlight the multifaceted nature of cancer treatment and its implications for pneumonia-related mortality.

Several studies have explored the use of machine learning models in the prediction and diagnosis of various diseases, including pneumonia and cancer. Machine learning-based variables with available and common clinically relevant characteristics can effectively predict survival in patients with community-acquired pneumonia (25). However, no studies have used this method to predict tumor death due to pneumonia. The development of the CatBoost predictive model is a significant advancement in our capacity to predict survival in cancer patients with pneumonia. The rigorous training and validation process of the model, which employs cross-validation to optimize hyperparameters, has resulted in a tool with exceptional predictive accuracy. The performance of the model, as evidenced by the ROC curves and AUC scores, surpassed that of traditional ML algorithms, indicating its potential superiority in clinical applications. The CatBoost model demonstrated consistent predictive performance across different time intervals, with AUC values of 0.8384 for 6-month survival, 0.8255 for 1-year survival, 0.8039 for 2-year survival, and 0.7939 for 3-year survival. This suggests that the model is robust and reliable for both short-term and long-term prognostic predictions.

External validation of the CatBoost model via an independent dataset further confirmed its robustness and generalizability. The confusion matrix analysis and assessment of clinical feature importance within the model reinforce the model’s efficacy and the critical role of surgery in prognosis, which is consistent with the findings from the Cox regression analysis.

In synthesizing these findings with the broader literature, it is clear that ML models can provide valuable insights into the complex interplay among cancer, pneumonia, and mortality. The success of the CatBoost model in predicting survival outcomes highlights the potential of ML to augment clinical decision-making and enhance patient management. CatBoost demonstrated superior performance in scenarios involving large datasets with numerous categorical variables, where traditional models like logistic regression and support vector machines require extensive preprocessing. Its ability to handle categorical data directly and model complex interactions made it particularly effective in predicting pneumonia-related mortality in cancer patients.

Future research should focus on expanding these models to include diverse populations and integrate them into clinical practice. This will enable the provision of more personalized care for cancer patients at risk of pneumonia-related mortality, ultimately aiming to improve survival rates and patient outcomes. The integration of ML with existing clinical tools and continuous refinement of these models will be crucial in addressing the intricate relationship between cancer and pneumonia, offering a more nuanced approach to patient care.

One study investigated the incidence of postoperative pneumonia (POP) in patients with the five most common cancers (gastric, colorectal, lung, breast, and hepatocellular carcinoma [HCC]) within 1 year of cancer surgery; the incidence rates of lung cancer were 8.0%, 1.8%, 1.0%, 0.7%, and 0.4%, respectively. In the multivariate analysis, older age, higher Charlson Comorbidity Index (CCI) scores, ulcer disease, history of pneumonia, and smoking were associated with the development of POP. Overall, the 1-year cumulative incidence of POP among the five most common cancers was 2%. Older age, higher CCI scores, smoking, ulcer disease, and a history of previous pneumonia increased the risk of POP in cancer patients (26). However, no study has examined the risk factors for tumor death from pneumonia. According to our Cox regression analysis, the top five factors affecting prognosis were surgery, stage, age, site, and sex, with surgery being the most significant factor in both the short-term (6 months and 1 year) and long-term (2 years and 3 years) prognostic models. A greater number of surgeries clearly increases patient survival in patients with digestive and endocrine tumors in both the short-term (6 months and 1 year) and long-term (2 and 3 years) prognostic models in our SHAP value analysis. Although the literature review did not directly address the specific relationship between surgery and a reduction in mortality in patients with oncologic pneumonia, we speculate that surgical interventions can be important in certain tumors to improve the prognosis of patients with a variety of medical conditions. Further research is needed to investigate the reasons for these findings.

### Strengths and limitations

The present study has several notable strengths along with some acknowledged limitations. One of the primary strengths of this study is the large sample size provided by the SEER database, which encompasses a substantial and diverse patient population. Rigorous data collection procedures within the SEER database further contribute to the reliability of the study findings.

Our CatBoost model, which is used to manage and guide general patient care, as well as personalized care, applied the following steps. First, a patient’s medical records were collected, including key information such as cancer type, surgical history, cancer stage, age, and sex. Patients were risk assessed via ML models to predict their likelihood of death from pneumonia. The model provides survival probability predictions on the basis of the specific circumstances of the patient. On the basis of the prediction results of the model, a personalized care plan is developed for each patient. For example, in high-risk patients, closer monitoring and prophylactic antibiotic therapy may be needed. As patient conditions change, new health data are continuously collected, and dynamic risk assessments are performed via ML models to adjust care plans in a timely manner. Using the model prediction results helps optimize the allocation of medical resources to ensure that high-risk patients receive the necessary medical attention and intervention. Interpretative analyses of models, such as SHAP analysis, are used to educate patients and families about the prognosis of the disease and why specific treatments or care measures are necessary. Targeted interventions are provided for patients on the basis of key influencing factors identified by the model, such as the impact of surgery on prognosis. For example, for patients with tumors of the digestive and endocrine systems, surgery may be recommended to improve patient prognosis. Collaboration among different healthcare professionals should be promoted to ensure comprehensiveness and consistency in patient care plans, especially in surgical and other critical treatment decisions. As new data accumulate and medical practice evolves, ML models are regularly updated and optimized to maintain their predictive accuracy and clinical relevance.

The study faced limitations due to the lack of detailed data in the SEER database, such as specific pathogens related to pneumonia, comorbidities, chemotherapy details, and specifics of radiotherapy. This granularity gap may hinder a comprehensive understanding of the relationship between cancer treatment and pneumonia risk. Additionally, potential misclassification of pneumonia or influenza in cancer patients with competing diagnoses and the inability to assess the risk of death from influenza or other respiratory diseases could affect the study’s accuracy. The study’s reliance on the SEER-9 registry and death certificates could introduce bias. These limitations suggest that this study can identify only associations, not causality, and more research is needed to identify cancer patients at greater risk of fatal respiratory infections and develop mitigation strategies. The reliance on the SEER database, while providing a large and diverse patient population, lacks detailed information on specific pathogens, comorbidities, and finer granularity on treatment modalities such as chemotherapy regimens and radiotherapy specifics. These gaps may limit the ability to fully understand the relationship between cancer treatments and pneumonia risk.

Future research should focus on incorporating more granular data on comorbidities, specific pathogens, and treatment modalities to enhance the predictive accuracy of machine learning models. Additionally, validating the CatBoost model across diverse populations and integrating it into clinical practice will be crucial for improving personalized cancer care and developing targeted interventions for high-risk patients.

## Conclusion

In conclusion, this study demonstrated the potential of machine learning in predicting the risk of death from pneumonia in patients with cancer. We believe that as technology further evolves and undergoes clinical validation, these models will provide robust support for clinical decision-making and ultimately improve patient outcomes. The integration of advanced predictive models into clinical practice has the potential to enhance personalized care for patients with cancer, enabling earlier interventions and improved management of pneumonia-related risks.

## Data Availability

The raw data supporting the conclusions of this article will be made available by the authors, without undue reservation.
